# Analysis of Bottlenecks in Experimental Models of Infection

**DOI:** 10.1371/journal.ppat.1004823

**Published:** 2015-06-11

**Authors:** Sören Abel, Pia Abel zur Wiesch, Brigid M. Davis, Matthew K. Waldor

**Affiliations:** 1 Department of Microbiology and Immunobiology, Harvard Medical School, Boston, Massachusetts, United States of America; 2 Division of Infectious Diseases, Brigham & Women’s Hospital, Boston, Massachusetts, United States of America; 3 Department of Pharmacy, University of Tromsø (UiT), The Arctic University of Norway, Tromsø, Norway; 4 Division of Epidemiology of Microbial Diseases, Yale School of Public Health, New Haven, Connecticut, United States of America; 5 Howard Hughes Medical Institute, Boston, Massachusetts, United States of America; Geisel School of Medicine at Dartmouth, UNITED STATES

## What Are Bottlenecks?

**Fig 1:**
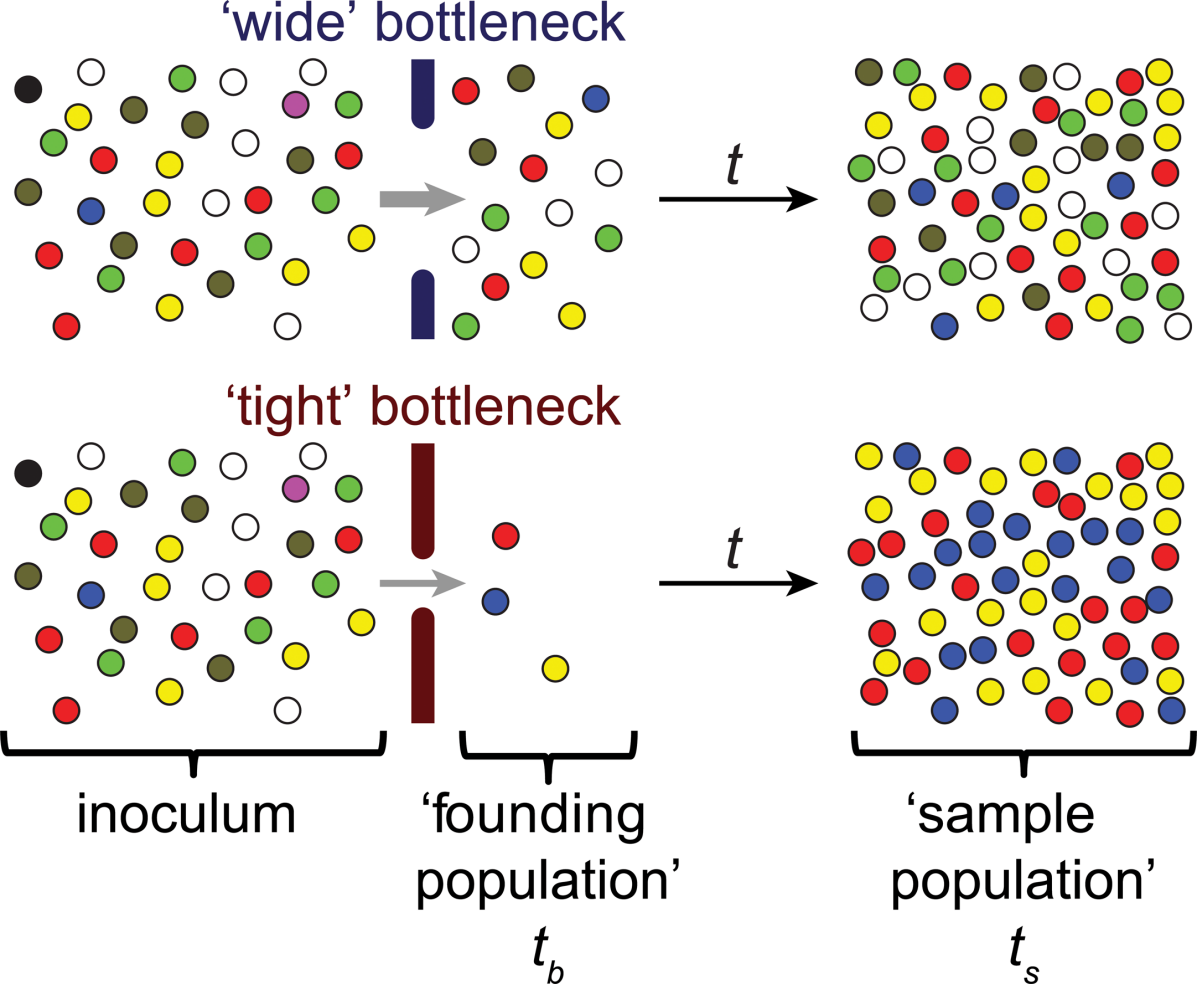
Schematic representation of the effect of bottlenecks on genetic diversity. Individual pathogens are shown as colored spheres; the colors represent distinguishable markers. The barriers to infection that constitute the bottleneck are shown by the solid bars and the size of the bottleneck is represented by the size of the gap between these bars. Bottlenecks are events that dramatically reduce the original population size, for example, the inoculum in infectious diseases. In the context of infection, the founding population consists of the pathogens that survive passage through the bottleneck and give rise to a population in a new environment, e.g., a new host or anatomical site. Often it is not feasible to sample directly after the bottleneck event (*t*_*b*_); instead, populations are sampled (at time *t*_*s*_) after the passage of time (t), represented by the black arrow. During this time, the founding population often replicates. Wide bottlenecks lead to limited loss of markers (e.g., the magenta and black spheres) and limited changes in the marker frequencies (e.g., over-representation of the blue and under-representation of the olive marker). In contrast, tight bottlenecks lead to stochastic loss of many markers and substantial changes in marker frequencies. These changes can be used to determine the magnitude of bottleneck events and the size of the founding population, even after the population size has increased, provided that the expansion has limited effect on the marker composition (i.e., markers are fitness neutral, and no additional genetic drift occurs).

The metaphor of a bottleneck has been used in a variety of fields to describe the critical constraints that limit a system’s performance or capacity. In biology, particularly in studies of population dynamics and evolution, the bottleneck concept is often used in reference to events that sharply limit population size [1]. Such events frequently produce stochastic changes in the genetic composition of a population (Fig 1), referred to as genetic drift. In extreme cases, population-reducing events can eliminate genotypes from a gene pool (Fig 1), even genotypes not associated with reduced fitness.

If the limited number of these surviving organisms found a population in a new environment, such as the colonization or infection of a host by microorganisms, those few organisms determine the genetic composition of subsequent generations, creating a “founder effect” within the post-bottleneck population [1]. These changes in genotype frequency are an important driver of evolutionary change and speciation. In infection biology, bottlenecks shape genetic diversity of epidemics and have been shown to have an important influence on the effect of recombination and horizontal gene transfer, as well as the evolution of drug resistance [2–6]. Furthermore, bottlenecks may reduce pathogen virulence and adaptability to new hosts, as they increase the rate at which attenuating mutations become fixed in a population [7,8].

Traditionally, population biologists have taken advantage of natural genetic variation to investigate transmission bottlenecks, e.g., during HIV transmission (reviewed in [9]). In addition, with pathogens that contain or accumulate high amounts of genetic diversity over a small timescale, such as HIV, it is possible to investigate bottlenecks within a single host [5]. However, many pathogens do not possess sufficient natural genetic variation for quantification of bottlenecks in this setting. Measuring bottleneck sizes in animal models allows experimental access to valuable information regarding the anatomical sites, sizes, and causes of population restrictions, which can provide key insights into the nature of host–pathogen interactions. Here, we focus on recent new methods that rely on introduction of artificial genetic variation to quantify bottleneck events during experimental infection, enabling more precise understanding of pathogen population dynamics.

## How Are Bottlenecks in Experimental Infections Measured?

In principle, the size of an infection bottleneck can be measured by simply counting the number of organisms (e.g., viral particles, bacterial colony forming units [CFU]) that survive a population-restriction process. However, enumeration of these organisms (sometimes referred to as the founding population) must occur prior to changes in their number, e.g., by replication or migration (Fig 1), and great experimental efforts and a large number of experimental animals are often required to pinpoint the sites and times of population constrictions [10]. Furthermore, when there is complex migration of the pathogen within the host, rather than a linear infection “pathway,” direct counting of individual infectious agents may not be feasible.

An alternative way to measure bottleneck sizes makes use of the stochastic changes in the genetic composition (diversity) of the infecting population that usually accompany population size reductions (Fig 1). This approach is employed in studies of natural populations, including certain viral pathogens, in which there is substantial genetic diversity [11–13]. However, for experimental studies of infection, often there is insufficient natural diversity in the inoculum for meaningful analyses. To circumvent this limitation, genetic variation has been introduced artificially into pathogen populations. Using inheritable, distinguishable markers that ideally do not alter pathogen fitness, changes in marker prevalence between the inoculum (i.e., the population prior to reduction by bottlenecks) and experimental samples can be used to estimate bottleneck sizes. Different genetic markers that have been used for this purpose include antibiotic resistances, lacZ+/lacZ-, fluorescent proteins, transposon insertions, restriction sites, and sequence tags [14–27]. The number of distinct markers is a major factor that limits the resolution of these assays, with a greater number of markers enabling greater resolution. When bottlenecks are very small, the assay limits of resolution are less of an issue; however, when there is a wide bottleneck, the size of the founding population cannot be accurately determined using a inoculum with low marker diversity [27]. Insertion of short DNA sequences into neutral loci in the genome, thereby generating wild-type isogenic tagged strains (WITS) [17], allows for easy creation of a large number of distinguishable strains. Sequence tags have been detected by a variety of methods, including hybridization, PCR, and most recently, DNA sequencing [25–27]. The availability of relatively inexpensive high-throughput sequencing that enables accurate quantification of a large number of sequence tags makes sequencing the current best approach to measure bottleneck sizes.

Several analytic approaches have been developed to identify bottleneck sizes based on differences in marker representation at two time points. These approaches include (i) probabilistic methods that analyze the stochastic loss of tagged strains [15,17,19,23,25], (ii) mathematical modeling [21,22], and (iii) population genetic approaches [18,27]. Analyses based on the presence or absence of individual marked strains are the most commonly performed because many experimental techniques can provide qualitative data about marker presence or absence. The major drawback of these analyses is their limited resolving power because the maximum bottleneck size that can be measured strongly depends on the number of distinguishable markers in the infecting population. Furthermore, since this approach is based on a stochastic model, relatively large numbers of repetitions are required for accurate measurements, usually necessitating the use of many experimental animals [25]. When more detailed knowledge about a pathogen’s behavior during infection is available, e.g., migration pattern in the host, mathematical models that explicitly describe this behavior can be used to estimate pathogen population size as well as the speed of migration between compartments [21]. These complex models can provide high-quality estimations but are dependent on knowledge of parameters that may not be available for less well-characterized systems. Finally, approaches that are based on population genetic theory [28,29] can be applied to estimate bottleneck sizes without knowledge of pathogen spatiotemporal dynamics within the host. These methods infer the bottleneck size by comparing allele frequencies (rather than just absence or presence of an allele) before and after bottleneck events (Fig 1). Two approaches can be distinguished: (i) those requiring data from several experiments, which yield the average bottleneck size across a series of hosts and are unable to distinguish between technical and biological variation between experiments [18,22], and (ii) those allowing bottleneck size determination in a single experiment with high accuracy [27]. The latter, recently developed method requires the use of a large number of tagged strains, but it allows the comparison of bottlenecks between individual hosts and, thereby, makes it possible to analyze the biological variance between hosts.

## What Are the Factors and/or Mechanisms That Restrict Pathogen Populations during Infections?

The nature of host bottlenecks, as well as pathogens’ strategies for circumventing them, continues to be a central topic in studies of host–pathogen interactions. All host defenses that counteract infection can be thought of as bottlenecks, as can some intrinsic features of the host environment. Impediments to infection can include physical barriers, innate and adaptive immune defenses, nutritional limitations, competing microorganisms, and niche availability (environments permissive for colonization and replication). The presence and mode of action of some barriers, such as the size of the pore in the squid light organ, low stomach pH, antimicrobial peptides, and low iron availability, have long been known. However, much recent and current research is devoted to uncovering new mechanisms of innate defense. Processes whereby the commensal microbiota restrict pathogen populations (e.g., competition for nutrients or colonization sites, or modulation of host immune development) are also only beginning to come into focus. Investigations of the specific mechanisms that mediate interbacterial competition and/or killing, such as Type VI secretion, will shed light on novel types of bottlenecks.

**Fig 2:**
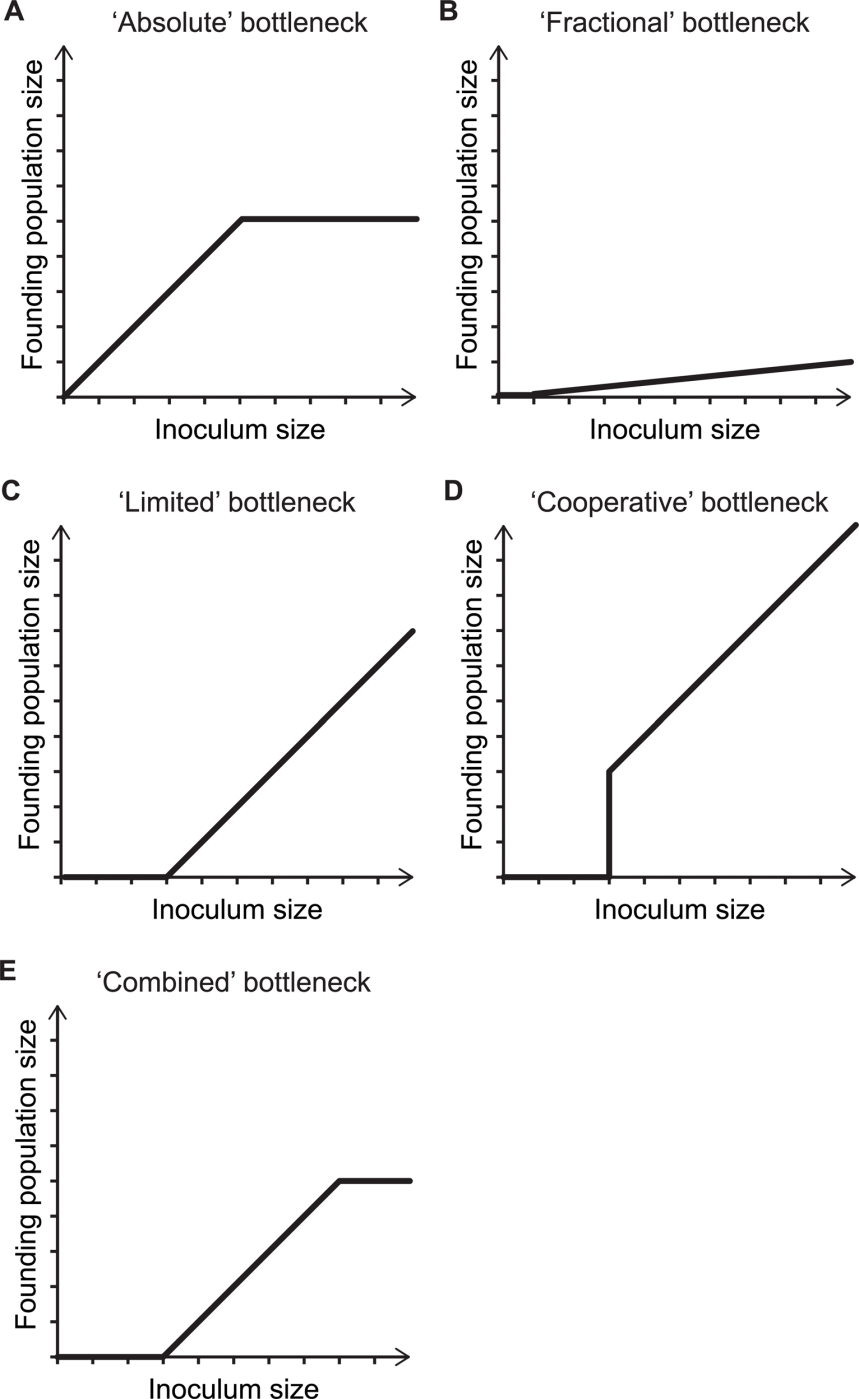
The mechanisms underlying bottlenecks shape the relationship between inoculum size and founding population size. Five conceptual examples of how the relationship between the inoculum size and founding population size changes with different types of bottlenecks. (**A**) An “absolute” bottleneck allows the unobstructed passage of organisms until its capacity is exhausted, thereby defining an upper limit on the number organisms that can pass. (**B**) During passage through a “fractional” bottleneck, a proportion of the inoculum does not survive. In this scenario, low inoculum sizes can occasionally give rise to infection, even if the expected bottleneck size is below one; however, for simplicity, in the graph, low inocula are set to a founding population size of zero. (**C**) With a “limited” bottleneck, a fixed amount of the inoculum is killed. (**D**) With a “cooperative” bottleneck, a population cannot pass through the bottleneck unless a sufficient number of organisms are present in the inoculum. Once the population size crosses this threshold, all organisms become competent for bottleneck passage. (**E**) In more realistic scenarios, diverse mechanisms of host defense collectively limit the founding population size.

On a more abstract level, host restrictions can either be toxic to pathogens or merely reflect limited resources that can only sustain a finite number of organisms. These different types of restriction will lead to different effects on the pathogen population. When there are finite resources available, there is an “absolute” limit to the size of the bottleneck; i.e., the bottleneck size is independent of the inoculum size, resulting in an upper limit to the size of the founding population (Fig 2A). In contrast, toxic host defenses can result in a “fractional” bottleneck, such that a proportion, rather than number, of the organisms survive (Fig 2B). For example, if a fraction of the inoculum is phenotypically resistant to the restricting conditions, then a definable percentage of the inoculum may survive. Alternatively, it is possible that host defenses can control a limited number of pathogens. In this “limited” bottleneck scenario, a high number of infecting organisms can exhaust host defenses, so that excess pathogens (above the saturating level) survive (Fig 2C). For fractional and limited bottlenecks, there is no upper limit to the size of the founding population; however, there is a lower limit to the inoculum size for establishment of infection. In addition, more complex biological mechanisms, e.g., quorum-sensing-regulated virulence [30,31], can result in complex inoculum size to founding population size relationships (Fig 2D). In reality, bottlenecks likely reflect a combination of such mechanisms (Fig 2E).

## Bottlenecks Can Confound Genome-Wide Analyses of Virulence and Transmission Studies

Bottlenecks can be a major technical challenge for the analysis of high-throughput (e.g., transposon-insertion sequencing) studies of genes required for infection. For example, genome-wide transposon insertion studies can be confounded by the existence of tight bottlenecks, which can limit the complexity of libraries that can effectively be analyzed. Moreover, when stochastic loss of transposon mutants due to bottlenecks overshadows the effects of fitness defects, many false positive classifications result [32]. Genetic drift that results from bottleneck events can also lead to pathogen populations that are very different between primary and secondary host. Such random differences may severely impact the accuracy and reliability of transmission chains constructed based on the genetic similarity between pathogen populations in different hosts [33]. Therefore, it is critical for high-throughput studies to account for bottleneck effects.

## Intriguing Questions That Can Be Addressed Using New Approaches for Measuring Bottlenecks

- Are bottleneck sizes constant during infection? Our recent work revealed that the size of the *Vibrio cholerae* founding population in the intestines of infected rabbits changes as infection progresses [27]. It will be fascinating to investigate the dynamics of the processes by which pathogens counteract host restrictions during infection.
- How and by what mechanisms does the composition of the microbiota (or co-infecting pathogens) modulate bottleneck sizes?
- Do virulence factors enable pathogens to overcome bottlenecks?
- Can we take advantage of bottlenecks to design improved regimens for antimicrobial administration or vaccination that limit emergence of resistance and have enhanced therapeutic efficacy?

Overall, the development of new tools to analyze infection bottlenecks creates a new way to understand host–pathogen interactions during infection.
